# Clinical management of metachromatic leukodystrophy cases identified in the German and Austrian Newborn screening pilots

**DOI:** 10.1186/s13023-026-04620-y

**Published:** 2026-10-04

**Authors:** Lucia Laugwitz, Pascal Martin, Nils Janzen, Sebastian Hegert, Thomas Neiße, Madeleine Wacker, Jan Kern, Nadja Kaiser, Christiane Kehrer, Annina-Clarissa Jaeger, Tom Launer, Philipp Juengling, Maximilian Zeyda, Vassiliki Konstantopoulou, Sebastian Graeter, Ursula Holzer, Peter Lang, Tim Flaadt, Barbara Plecko, Lukas Neubauer, Daniela Karall, Thomas Zöggeler, Stephan T. Hold, Petra Oliva, David Kasper, Stefanie Beck-Woedl, Tobias Haack, Hendrik Rosewich, Samuel Groeschel

**Affiliations:** 1 Neuropediatrics, General Pediatrics, Diabetology, Endocrinology and Social Pediatrics, University of Tübingen, University Hospital Tübingen, Tübingen, Germany; 2 Department of Neurology and Epileptology, Hertie Institute for Clinical Brain Research, University Hospitals Tübingen, Tübingen, Germany; 3 Screening-Labor Hannover, Hannover, Lower Saxony Germany; 4 Hannover Medical School, Department of Clinical Chemistry, Hannover, Lower Saxony Germany; 5 Austrian Newborn Screening, Comprehensive Center for Pediatrics, Department of Pediatrics and Adolescent Medicine, Medical University of Vienna, Vienna, 1090 Austria; 6 Department of Pediatric Oncology, University of Tübingen, University Hospital Tübingen, Tübingen, Germany; 7 Department of Pediatrics and Adolescent Medicine, Division of General Pediatrics, Medical University of Graz, Graz, Austria; 8 Clinic for Pediatrics, Division of Inherited Metabolic Disease, Medical University of Innsbruck, Innsbruck, Austria; 9 ARCHIMEDlife GmbH, Vienna, Austria; 10 Institute for Medical Genetics and Applied Genomics, University Hospital Tübingen, Tübingen, Germany; 11 Department of Pediatric Neurology, University Children’s Hospital Göttingen, Göttingen, Germany; 12 German Center for Child and Adolescent Health (DZKJ), Partner Site Göttingen, Göttingen, Germany

**Keywords:** Metachromatic leukodystrophy, Newborn screening, Autologous hematopoietic stem and progenitor cell gene therapy, Lysosomal storage disease

## Abstract

**Background:**

Metachromatic leukodystrophy (MLD) is a rapidly progressive leukodystrophy that leads to severe disability and early death if untreated. Autologous hematopoietic stem and progenitor cell gene therapy (HSPC-GT, atidarsagene autotemcel, arsa-cel) for early onset subtypes and allogenic hematopoietic stem cell transplantation (HSCT) for late onset disease substantially alters disease progression for early onset disease when administered before symptom onset, creating a strong rationale for newborn screening (NBS). At the same time, NBS technique for MLD in dried blood spots has recently been demonstrated to be robust and highly accurate. The aim was to give real-world results from the world’s first NBS pilots for clinical management and treatment of identified children.

**Methods:**

Between September 2021 and July 2025, 359,282 newborns underwent NBS for MLD in two different laboratories in Germany and Austria using a three-tier algorithm integrating sulfatide quantification, arylsulfatase A (ARSA) activity measurement, and *ARSA* sequencing. Screen-positive infants underwent a predefined care pathway including standardized confirmatory diagnostics, genotype-based and biochemical prediction of disease onset, clinical assessment and management guiding early treatment and surveillance at the qualified treatment center (QTC) in Tübingen.

**Results:**

Nine newborns screened positive and all were confirmed to have MLD (detection rate approximately 1 per 40,000). Based on genotype and leukocyte ARSA enzyme activity, disease onset prediction was possible in all of them. Seven infants were classified as having pre-symptomatic early-onset MLD and were referred for HSPC-GT. All treated infants showed preserved neurological function up to 30 months after treatment (median 18.5 months). Two infants predicted to develop late-onset MLD entered structured surveillance for treatment with HSCT and have remained clinically asymptomatic for CNS signs of disease. No false-positive or known false-negative results were observed.

**Conclusion:**

These results from our pilot programs demonstrate that NBS enables reliable early identification of MLD and support streamlined care pathways leading to timely intervention. Importantly, this study provides real-world evidence illustrating that NBS for MLD can enable timely, pre-symptomatic treatment and structured surveillance within standard national healthcare systems. These findings further substantiate the value of NBS for MLD at a critical moment as several countries consider national implementation of MLD screening.

**Supplementary Information:**

The online version contains supplementary material available at https://doi.org/10.1186/s13023-026-04620-y.

## Introduction

Metachromatic leukodystrophy (MLD) (MIM#250100) is a rare lysosomal storage disorder caused by biallelic clinically relevant variants in *ARSA* (MIM*607574) encoding the lysosomal enzyme arylsulfatase A (ARSA) [1–3]. ARSA deficiency leads to a progressive accumulation of sulfatides, in particular in the central and peripheral nervous system, resulting in demyelination and neurodegeneration [1]. The overall birth prevalence of MLD in Europe is approximately 1 in 40,000 to 1 in 100,000 [2]. Although disease onset spans infancy to adulthood, early-onset forms (i.e. late infantile and early juvenile) are most common and are associated with more rapid neurodevelopmental regression, severe disability, and early death if untreated [4]. For these children, irreversible neurological injury begins early in life, making timely intervention critical. Late onset MLD (i.e. late juvenile and adult) may present later in childhood, adolescence, or adulthood, with a more gradual progression of severe neurological or psychiatric symptoms [4, 5].

Until recently, therapeutic options for early-onset MLD were limited to supportive care and, in late onset cases diagnosed in an early symptomatic or pre-symptomatic stage, allogeneic hematopoietic stem cell transplantation (HSCT) [6–8]. The development of autologous hematopoietic stem and progenitor cell gene therapy (HSPC-GT, atidarsagene autotemcel, arsa-cel) has fundamentally altered the treatment landscape for early onset MLD [9, 10]. When administered before the onset of clinical signs, HSPC-GT in early onset MLD and HSCT in late onset MLD are associated with preservation of early neurodevelopment and substantial modification of disease course [6–10]. However, treatment efficacy is highly time dependent: once neurological symptoms emerge, therapeutic benefit is markedly reduced [9]. This narrow therapeutic window means that for most children diagnosed with MLD, the diagnosis comes too late for currently available interventions [11]. Long-term outcomes are highly encouraging, with infants maintaining near-normal development when treated pre-symptomatically [9, 10]. Newborn screening (NBS) provides a population-based strategy to identify infants with MLD pre-symptomatically and before the onset of irreversible neurological injury [12]. Over the past decade, biochemical screening approaches for MLD have been systematically developed and validated in retrospective studies [13–15]. Prospective screening pilot programs have demonstrated that MLD screening can be integrated into established NBS infrastructures with high analytical performance [16, 17]. These advances have driven growing international momentum, with several countries initiating or expanding pilot programs and some incorporating MLD into national screening panels [18].

However, the availability of treatment options and screening alone does not ensure improved clinical outcomes. As a rapidly emerging field of science, gaps in evidence remain. For NBS for MLD this primarily relates to the downstream consequences of population-based detection: data are scarce on the real-world clinical management of infants identified through NBS for MLD, including confirmatory diagnostic workflows, prediction of disease onset, decision-making around presymptomatic treatment, and the feasibility of delivering time-sensitive interventions within routine healthcare systems. Equally important is our understanding how infants predicted to develop late-onset disease can be monitored safely over time without exposing families to unnecessary intervention or harm before they are eligible for treatment [19, 20].

In this work, we report the first larger clinical experiences from prospective NBS pilot programs for MLD in Germany and Austria. We evaluate previously established care pathways from screening to confirmatory diagnosis, consensus-based prediction of disease onset, and subsequent clinical management. We further report the clinical care pathway of pre-symptomatic infants with MLD who underwent HSPC-GT and those managed through structured surveillance before HSCT. Taken together, these findings will offer preliminary evidence for the feasibility and potential clinical impact of NBS-enabled care for MLD in routine pediatric healthcare practice. This study advances the field by providing the first prospective real-world data on early clinical trajectories after NBS for MLD, including confirmatory diagnostic pathways, treatment decisions and outcomes.

## Methods

### Pilot program design

Prospective NBS pilot programs for MLD were conducted in North-West Germany and nationwide in Austria between September 2021 and July 2025 through coordinated collaborations involving the Screening Laboratory Hannover, the Medical University of Vienna, and ARCHIMEDlife.

German newborns born in the capture area of the Screening Laboratory Hannover were enrolled under an Institutional Review Board-approved protocol (Ärztekammer Niedersachsen; BO/39/2021). Austrian screening was conducted under approval from the Ethics Committee of the Medical University of Vienna (1961/2022). Further biochemical and clinical investigations of infants identified through screening were approved by the local ethics committee of the Medical Faculty of the University of Tübingen, Germany (948/2018BO2). All participants, or their respective legal guardians, provided written informed consent for participation and publication in accordance with the Declaration of Helsinki. Clinical trial number: not applicable.

The Hannover cohort includes three screen positive infants previously reported in Laugwitz et al. 2024 (index 1 to 3) [16], with extended follow-up and additional cases included here. The Austrian cohort has not been reported before. All analyses reported in the present study were prospectively defined and conducted within the approved protocols.

The primary outcome of the pilot programs was the detection rate of confirmed MLD cases, with an expected birth prevalence of approximately 1 per 40,000 to 100,000 newborns based on European prevalence estimates [3].

The clinical data collection for this study was completed in December 2025. Current age and clinical status for every infant, including those followed at the referring centres in Graz and Innsbruck, were ascertained as of this date; current age was calculated from date of birth, and status updates for infants managed outside Tübingen were obtained by the coordinating QTC through direct communication with the local treating centres.

### Screening algorithm

NBS for MLD was implemented using a three-tier screening strategy, as previously described [15, 16, 21]. The screening workflow evolved during the pilot phase to optimize analytical performance and minimize false-positive results. Since early 2024, the fully standardized three-tier algorithm has been applied to dried blood spots (DBS) obtained for routine NBS in both Germany and Austria, and consists of:

1. quantification of sulfatides (C16:0 and C16:1-OH) in dried blood spots (DBS),
2. measurement of ARSA enzymatic activity in DBS, and
3. next generation sequencing of *ARSA*, *SUMF1*, and *PSAP* in DBS.

Detailed analytical methods, assay cut-offs, and quality control procedures have been reported previously [15, 16, 21].

### Clinical management pathway

**Fig. 1:**
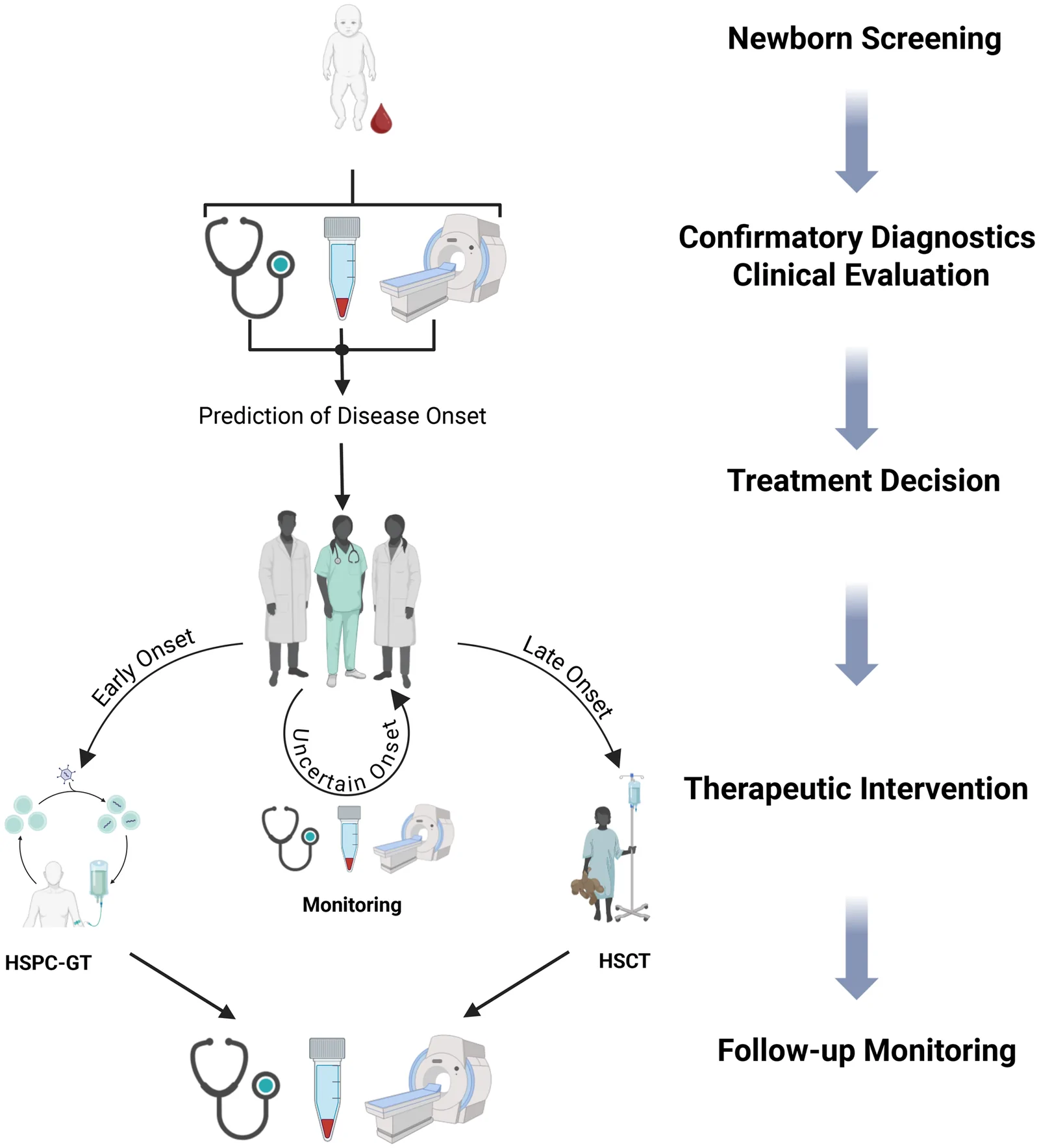
Clinical management pathway following newborn screening for metachromatic leukodystrophy (MLD). The diagram illustrates the stepwise clinical workflow following NBS for MLD. After detection via three-tiered screening of dried blood spots, confirmatory diagnostics and clinical evaluation are initiated to predict disease onset. Confirmatory testing includes ARSA activity in leukocytes, urinary sulfatide levels and genetic testing of the infant and both parents. Based on the predicted subtype – early, late, or uncertain onset - a tailored treatment decision is made. Presymptomatic early-onset cases are eligible for HSPC-GT within the first year of life, while late-onset forms are scheduled for HSCT at pre-school ages. In cases with late or uncertain onset, longitudinal surveillance is mandatory. All affected individuals continue under structured follow-up to assess long-term outcomes

A central aim of the pilot programs was not only case detection but also the prospective evaluation of a standardized, consensus-based care pathway following a positive NBS result. Management of infants identified through NBS followed a predefined, standardized care pathway aligned with European expert consensus recommendations (Fig. 1) [20]. After confirmation of the diagnosis of MLD, families received multidisciplinary counselling regarding disease characteristics, prognosis, and available management options. Care was coordinated by the QTC in Tübingen in collaboration with metabolic pediatricians, pediatric neurologists, clinical geneticists, transplant physicians, and allied health professionals. Psychosocial support and access to patient advocacy resources were offered throughout the diagnostic and management process in accordance with consensus guidance. Cases were discussed within the treatment eligibility panel of the MLDInitiative (MLDi) [22].

### Confirmatory diagnostics and molecular assessment

For all screen-positive infants, families were informed by MLD-specialized treatment centers in line with European recommendations (i.e. the QTC in Tübingen, Germany, and medical centers in Graz and Innsbruck, Austria), and infants were referred promptly for confirmatory diagnostic evaluation. Confirmatory testing was coordinated through specialized centers in collaboration with the QTC at the University Hospital Tübingen, designated as a QTC for Germany and Austria. Confirmatory diagnostics included measurement of ARSA activity in leukocytes [23, 24] and repeated quantification of urinary sulfatide levels using liquid chromatography-tandem mass spectrometry [25]. Genetic sequencing was repeated using freshly obtained EDTA blood samples from the infant and both parents to confirm biallelic *ARSA* variant localization. Following biochemical confirmation, *ARSA* variants were classified according to American College of Medical Genetics and Genomics (ACMG) [26].

### Clinical evaluation

Baseline clinical evaluation at the QTC included a comprehensive neuropediatric examination and assessment of developmental milestones. Instrumental investigations comprised brain magnetic resonance imaging, nerve conduction velocity studies, peripheral nerve ultrasound, acoustic evoked potentials, and gallbladder ultrasound. The scope and timing of assessments were adapted to age and clinical context while adhering to consensus recommendations [20].

### Prediction of disease onset

Each infant was then classified into an MLD predicted-onset category, based primarily on genotype: early onset (late infantile or early juvenile) versus late onset (late juvenile or adult). Prediction was further supported by repeated measurement of residual ARSA activity in leukocytes [24]. Published consensus criteria for phenotype prediction in pre-symptomatic infants were applied [20]: In brief, infants carrying two predicted protein truncating variants (pPTVs) in *ARSA*, known variants associated with late infantile onset or who exhibited a residual ARSA activity in leukocytes of less than 1% on repeated measurements were predicted to develop the late infantile form of MLD [24]. In line with European consensus recommendations, infants carrying a pPTV *in trans* with a clinically relevant missense variant were classified as early-onset MLD (i.e. late-infantile or early-juvenile MLD). Because in some cases these two early-onset courses cannot reliably be separated by genotype alone, such infants were grouped together as early onset for treatment decisions. Newborns with genotypes consistently associated with late onset disease such as c.542T > G (p.Ile181Ser) *in trans* with any other clinically relevant *ARSA* variant or c.1283C > T (p.Pro428Leu) in homozygous state were predicted as late onset, recognizing that distinguishing late juvenile versus adult onset is not possible with genotype alone [20]. Newborns with other well-documented *ARSA* genotypes reported in the literature were classified accordingly.

### Treatment decision

All cases were reviewed in multidisciplinary European expert rounds of MLDi. Eligibility for treatment with HSPC-GT (arsa-cel) was assessed according to European Medicines Agency criteria [27]. Consensus decisions regarding treatment, timing, and monitoring were reached by a multidisciplinary expert panel. Infants predicted to develop early-onset MLD were referred for gene therapy within the first 12 months of life. Infants predicted to develop late-onset disease or for whom disease onset was uncertain, were enrolled in a structured long-term surveillance program, which includes regular neurological examinations, developmental assessments, and periodic neuroimaging and neurophysiological studies. In practice, infants under surveillance were reviewed clinically with developmental assessments, brain MRI and neurophysiological studies performed at the age-specific intervals of the surveillance schedule published in the European expert recommendations [20, 28] or whenever clinically indicated; emergence of white-matter change on MRI, neurophysiological progression, or biomarker change prompted expedited re-evaluation of the treatment decision. Escalation to therapeutic intervention during surveillance followed consensus recommendations and was based on emerging clinical, neurophysiological, imaging, or biomarker evidence of disease activity. Outcome data were collected prospectively.

### Statistical methods and graphics

Descriptive statistics were applied to summarize the screened population, biochemical and genetic findings, and diagnostic outcomes. Illustrations were generated using Biorender and the Python package *matplotlib* (version 3.10.3). Descriptive statistics were calculated using Python (version 3.13.0) primarily utilizing the packages *pandas* and *numpy*.

## Results

### Screening yield and case ascertainment

Between September 2021 and July 2025, a total of 359,282 dried blood spot samples were analyzed across NBS pilots in Germany and Austria. Nine newborns screened positive and were subsequently confirmed to have MLD, corresponding to an observed detection rate of 2.51 per 100,000 screened newborns (one in 39,920). Because birth prevalence reflects the stable genetic frequency of MLD, whereas the number of cases detected per year depends on the birth rate of the screened region, we report this figure as an observed detection rate rather than as an incidence estimate. No false-positive recalls occurred during the study period. During the study period, no additional infants diagnosed with MLD within the screening catchment areas were reported to the QTC in Tübingen.

### Management timeline

**Fig. 2:**
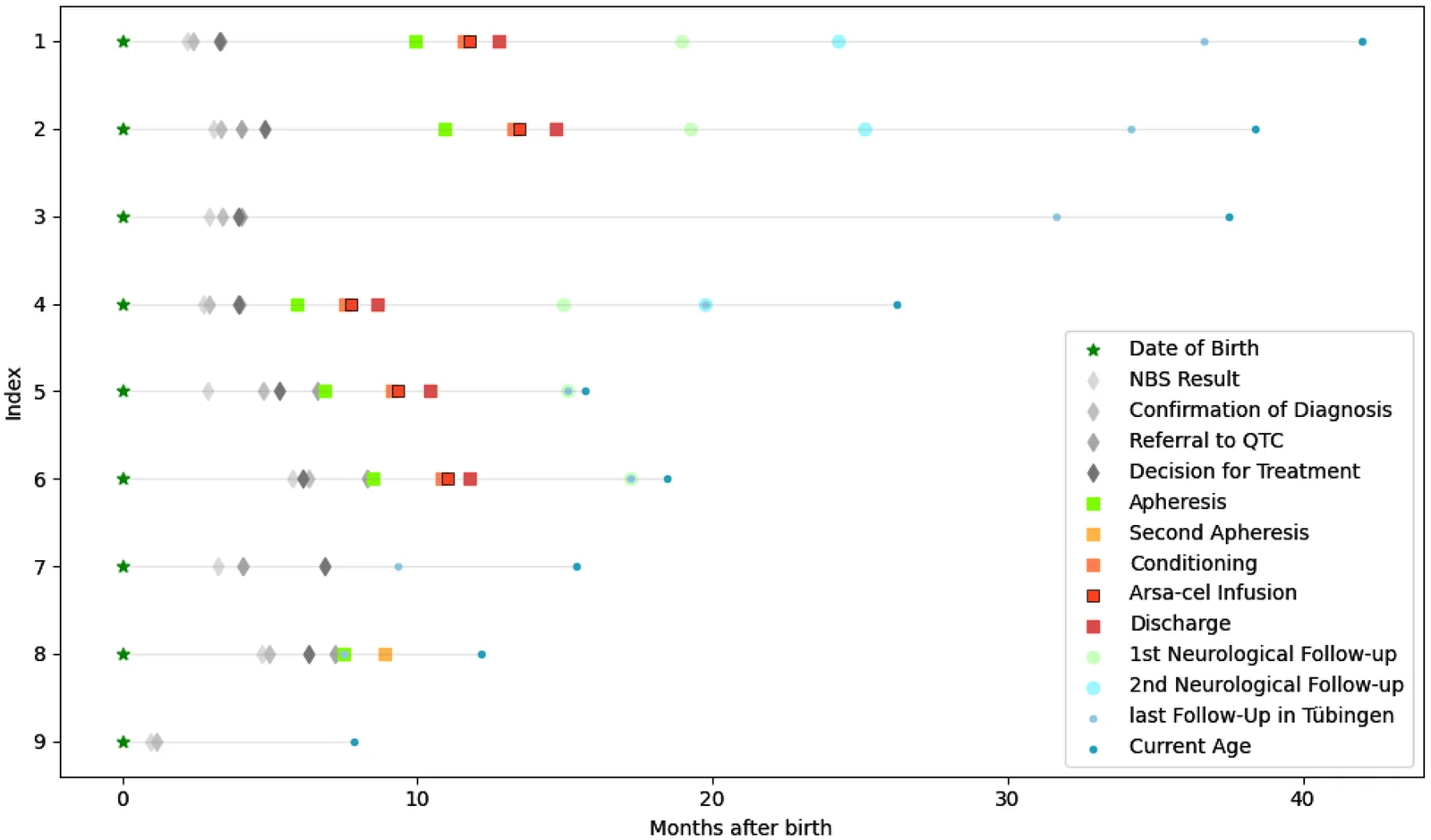
Timeline of patient management. Timeline of key clinical milestones for nine patients identified by NBS for MLD, showing the course from birth through current age. Each horizontal line represents an individual patient, with markers indicating specific events such as screening results, diagnosis confirmation, treatment decisions, medical procedures (apheresis, conditioning, arsa-cel infusion), discharge, and neurological follow-ups. The x-axis measures months after birth, while the legend identifies each event type by symbol and color. Event metrics are listed in Supplement table S2. For the two infants predicted to develop late-onset MLD (index 3 and index 9), the management decision was enrolment in structured surveillance with HSCT planned in the pre-school period

Screen-positive results for the nine index cases identified in the NBS studies were communicated to the designated QTC in Tübingen at a median age of 3 months (range 1.0 - 5.8 months) following NBS sample collection at birth (Fig. 2). The upper end of this range reflected the evolving pilot workflows in different participating laboratories rather than the final steady-state pathway: in the early phase, second- and third-tier confirmatory testing and, in the German cohort, the transfer of samples between the collaborating laboratories added variable processing time before a definitive screen-positive result could be communicated to the QTC. With the fully standardized algorithm now in place, these intervals are shortened substantially. In accordance with established European NBS recommendations, families were contacted within one week of NBS result receipt by an designated MLD expert center. In eight cases, initial disclosure was conducted by the MLD expert center. In one case, families were contacted directly by the screening laboratory owing to a miscommunication with the referring laboratory; this represented a protocol deviation. This deviation concerned only the communication pathway for the initial result disclosure; the infant subsequently underwent the identical confirmatory-testing, phenotype-prediction, and treatment pathway as all other cases. It therefore had no bearing on case ascertainment, diagnostic accuracy, subtype classification, or clinical outcome, and does not affect the reported detection rate. All families were offered rapid access to the QTC through video or telephone consultation or in-person outpatient visits within one week after notification of positive NBS results. Confirmatory diagnostic testing was completed within a median of 0.3 months after recall (range 0.2 - 1.9 months). Comprehensive clinical evaluations at the expert center were conducted within a median of 1.0 month following diagnosis (range 0 - 2.3 months), with timing adjusted according to predicted disease subtype. All cases were subsequently reviewed by a multidisciplinary treatment eligibility panel convened by the MLDi within 1 to 4 months after recall. For infants predicted to develop late-infantile disease, panel review was prioritized and scheduled within 1 to 2 weeks after completion of confirmatory diagnostics. Treatment recommendations were reached by consensus in eight of nine cases at the initial panel meeting. One case (index 7) required additional diagnostic clarification and repeat review before final consensus was achieved (Supplement, Case reports). Subsequent clinical assessments at the QTC were completed within 0 to 3 months for all infants and within 4 to 6 weeks for those with predicted early-onset disease. Stem cell apheresis was performed a median of 2.2 months after treatment decision (range 1.2 - 6.6 months), corresponding to a median of 3.6 months after NBS results (range 2.7 - 7.9 months). In one case (index 8), apheresis was repeated because the initial drug product did not meet release criteria, consistent with standard manufacturing protocols. HSPC-GT infusion was administered a median of 2.5 months after apheresis (range 1.8 - 2.5 months). Following treatment, infants were discharged a median of 1.0 month after infusion (range 0.8 - 1.2 months; Fig. 2).

### Confirmatory diagnostics and molecular findings

All nine infants classified as screen positive were confirmed to have a diagnosis of MLD through biochemical and molecular testing (Table 1). Each infant harbored biallelic clinically relevant variants in *ARSA*, accompanied by markedly reduced ARSA activity in leukocytes and elevated sulfatide excretion in urine (Table 1; Figure S1). To account for biological variability, leukocyte ARSA activity and urinary sulfatides were assessed repeatedly at multiple time points (Table 1; Figure S1). Although some interday variability was observed, all repeated measurements remained consistently within the pathological range. Genotypes spanned the known phenotypic spectrum of MLD and included both previously reported pathogenic and novel variants. Three previously unreported *ARSA* variants were identified, including a splice donor variant, a frameshift and a missense variant. Based on ACMG criteria, these were classified as likely pathogenic, supported by functional biochemical confirmation (Table 1). Inclusion of the third-tier genetic testing in the NBS algorithm was critical for screening specificity. No infants with pseudodeficiency alleles or isolated heterozygous variants in *ARSA*, or variants in *SUMF1* or *PSAP* fulfilled criteria for a positive screen, and no biochemical mimics of MLD were identified. Together, the combined biochemical-genetic approach in the screening and confirmatory testing algorithm enabled unambiguous diagnosis in all cases and avoided variants of uncertain clinical significance as standalone findings.

**Table 1:** Confirmatory diagnostics of newborns identified

Index	Genotype (HGVS, GRCh38)	ACMG Class	ClinVar	**ARSA activity in leukocytes** [24] Normal range: 1.5 to 6.1 E_514_ nm/10^6^ cells	Urinary Sulfatides Normal range: <300 nmol/l	Predicted Onset
1	ARSA:c.[412C > T];[1283C > T],p.[(Pro138Ser)];[(Pro428Leu)]	5;5	P [1]; P	0.007 ± 0.008 (*n* = 4)^a^	Pathologically elevated^b^	Early juvenile
2	ARSA:c.[465+1 G > A];[1283C > T],p.[(?)];[(Pro428Leu)]	5;5	P [1]; P	0.048 ± 0.013 (*n* = 6)	549.823 ± 265.427 (*n* = 4)	Early juvenile
3	ARSA:c.[465+1 G > A];[542T > G],p.[(?)];[(Ile181Ser)]	5;5	P;P	0.034 ± 0.029 (*n* = 8)	3216.248 ± 2723.202 (*n* = 4)	Late juvenile/ adult
4	ARSA:c.[465+1 G > A];[465+1 G > A],p.[(?)];[(?)]	5;5	P.;P	0.014 ± 0.017 (*n* = 4)^a^	1245.992 ± 892.734 (*n* = 4)	Late infantile
5	ARSA:c.[465+1 G > A];[899del],p.[(?)];[(Leu300Cysfs*29)]	5; 5	P;n.r.	0.035 ± 0.034 (*n* = 5)^a^	1960.298 ± 1045.793 (*n* = 6)	Late infantile
6	ARSA:c.[931 G > A];[931 G > A],p.[(Gly311Ser)];[(Gly311Ser)]	4;4	LP;LP	0.022 ± 0.018 (*n* = 4)^a^	2324.697 ± 753.985 (*n* = 3)	Late infantile
7	ARSA:c.[465+1 G > A];[494C > T];p.[(?)];[(Pro165Leu)]	5;3 ->4	P;n.r.	0.087 ± 0.018 (*n* = 6)	1933.71 ± 1679.955 (*n* = 8)	Early juvenile
8	ARSA:c.[684+1 G > A];[1223_1231del],p.[(?)];[(Val408Serfs*21)]	5;5	n.r.;P	0.015 ± 0.022 (*n* = 6)^a^	1149.3 ± 1068.438 (*n* = 2)	Late infantile
9	ARSA:c.[1283C > T];[1283C > T],p.[(Pro428Leu)];[(Pro428Leu)]	5;5	P;P	0.047 ± 0.042 (*n* = 4)	1680.425 ± 1254.938 (*n* = 2)	Late juvenile/ adult

n.r., not reported; P, pathogenic; LP, likely pathogenic; ^a^below the predictive threshold of <1% of mean activity in healthy controls according to prior reports [19, 21], suggestive for early onset^.b^ no quantification possible due to lack of stored biomaterial, sulfatides were measured qualitatively instead. n denotes the number of independent measurements obtained at separate time points for leukocyte ARSA activity and for urinary sulfatides, respectively; values are given as mean ± standard deviation

### Clinical baseline evaluation

Baseline clinical assessments were completed in all infants at the QTC in Tübingen prior to treatment or enrolment into surveillance (Supplement Table S2, Case Reports). At initial evaluation (between 3 and 6 months of age), neurological examination and developmental assessments were unremarkable in all cases, with achievement of age-appropriate milestones reported by caregivers and primary care physicians.

MRI of the brain was conducted in eight infants and showed no imaging evidence of leukodystrophy-related white matter changes, for one child with late onset-MLD the first MRI evaluation is planned but not yet performed. MRI severity scores were 0 in all cases (Supplement Table S1). A normal baseline MRI documents the absence of established white-matter disease and provides an individual reference against which the shortest interval change can be detected during surveillance. Gallbladder ultrasound revealed no gallstones or premalignant lesions. Mild gallbladder wall thickening or sludge was observed in two infants without associated clinical signs (Supplement Table S1).

Neurophysiological studies demonstrated greater variability. Nerve conduction studies were within age-adjusted reference ranges in three infants, while six infants showed evidence of demyelinating peripheral neuropathy, including infants predicted to develop both early- and late-onset disease. Peripheral nerve ultrasound was normal in all infants examined. Auditory evoked potentials were abnormal in two cases, showing prolonged latencies without corresponding clinical hearing impairment (Supplement Table S1). Overall, baseline evaluations confirmed the absence of overt neurological disease at the time of diagnosis while identifying subclinical abnormalities in selected instrumental assessments. Even though MRI and NCV studies require sedation, we considered those as important individual baseline investigations in order to detect subclinical onset of disease and to serve for comparison during follow-up.

### Prediction of disease onset

Based on the consensus criteria, the patients were stratified into predicted phenotypic subtypes:

**Late infantile MLD**: Out of the nine cases, four infants were predicted to have late infantile onset MLD. These infants harbored truncating ARSA variants known to abolish enzyme function (Table 1). Besides the common splice site variant c.465+1 G > A and known frameshift variant (c.1223_1231del), a novel splice donor variant (c.684+1 G > A) and a novel frameshift variant were identified (c.899del). Aligning with ACMG criteria these novel pPTVs were classified as likely pathogenic (class 4). Biallelic pPTVs strongly indicated symptom onset before 30 months of age and the most aggressive disease course without intervention (Table 1). All four had very low mean ARSA activity in leukocytes (<1% of healthy controls) and pathologically elevated urinary sulfatide excretion.

(Table 1, Figure S1)

**Early juvenile MLD:** Three infants were classified as early juvenile onset forms based on genotype data. Each of these cases carried one missense variant *in trans* with a pPTV in *ARSA* (Table 1). Two of these cases (index 1 and 2) carried *ARSA* genotypes previously reported multiple times in the literature, with mostly early juvenile onset (i.e. >80% of cases with comparable genotypes), and only few late juvenile cases in literature [24]. Their leukocyte ARSA activities, while clearly deficient, were somewhat higher than those of the late infantile cohort (Supplement Figure S1). Based on the European consensus criteria these cases were classified as early juvenile despite recognizing the variable onset due to certain genotypes even amongst siblings [29].

The third case (index 7) harbored one missense variant of uncertain significance (VUS, class 3) *in trans* with a well-known pathogenic pPTV. The prediction of early juvenile onset was based on data from individuals with comparable *ARSA* genotypes (Supplement, Case reports); the demyelinating neuropathy confirmed subclinical disease involvement but was not used as a specific discriminator of early-juvenile versus late-onset CNS disease. Based on the reduced ARSA activity in leukocytes and elevated urinary sulfatides in urine the variant was subsequently re-classified as likely pathogenic (class 4). Index 7 was referred for HSPC-GT based on an unanimous expert decision during MLDi treatment panels.

**Late Onset MLD:** two infants were classified as late-onset MLD based on the well reported genotype applying to European consensus recommendations. One infant harbored the c.542T > G *ARSA* variant exclusively present in late onset cases *in trans* with a pathogenic pPTV (index 3) [5, 24]. One infant carried the c.1283C > T variant in the homozygous state, also associated with a disease onset later than 6 years of age (index 9) [24].

### Treatment allocation and course

The multidisciplinary treatment eligibility panel achieved a unanimous consensus regarding the respective therapeutic intervention, timing of intervention and monitoring schedule before and after treatment aligned with European consensus recommendations. All infants predicted to develop early-onset disease - the four with predicted late-infantile and the three with predicted early-juvenile MLD (Table 1; Sect. “Prediction of disease onset”) - were referred for HSPC-GT. At the time of reporting, five infants had received treatment, one infant was awaiting infusion following apheresis, and one infant was undergoing treatment preparation (Fig. 2). Treatment was initiated at a median age of 11.0 months (range 7.8 - 13.4 months), corresponding to a median of 6.5 months after receipt of screening results. Conditioning and HSPC-GT infusion were generally well tolerated. Catheter-associated adverse events occurred in three infants and resolved without long-term sequelae (Supplement Table S1, Case Reports). No serious adverse events related to the gene therapy product were observed. Two infants predicted to develop late-onset disease were enrolled in structured longitudinal surveillance planning to undergo HSCT according to the care pathway [20].

### Early outcomes following HSPC-GT

Early clinical outcomes following presymptomatic identification and treatment were encouraging and in line with results from the previous treatment trial [9]. Follow-up after HSPC-GT ranged from 6.3 to 30.2 months (median 18.5 months). Neurological examinations during follow-up did not reveal clinical signs of disease progression in treated infants. Four of five treated infants demonstrated age-appropriate cognitive, motor, and language development at last assessment (Supplement Table S1). One infant with predicted late-infantile disease showed mild motor delay at 12 months post-treatment in the context of otherwise continuous developmental progress without signs of stagnation (Supplement, Case Reports, index 4). Brain MRI performed up to 24 months post-treatment showed no leukodystrophy-related white matter changes in any treated infant. Among the treated infants with serial nerve-conduction data, trajectories were variable, with progression in two infants and stability or partial improvement in the remainder. Of note, the treated child with mild developmental delay revealed clearly progressing demyelinating neuropathy (Supplement, Case Reports, index 4). Leukocyte ARSA enzyme activity remained within normal or supranormal ranges following treatment (Figure S1).

### Outcomes under structured surveillance

The two infants predicted to develop late-onset MLD remained clinically asymptomatic at last follow-up (age range 7.85 to 37.49 months). Serial neurological examinations and developmental assessments were within age-appropriate ranges. Neurophysiological findings showed mild abnormalities without progression, and no infant met criteria for therapeutic intervention during the observation period (Supplement Table S1). HSCT is scheduled for both children in the pre-school period.

## Discussion

These findings have direct implications not only for the clinical management of infants identified through NBS for MLD, but also for the ongoing evaluation and implementation of MLD screening within national programs. In particular, they provide real-world evidence that population-based screening can be effectively translated into timely, coordinated, and clinically actionable care pathways within established healthcare systems.

Beyond demonstrating feasibility, our results address key questions that are central to current policy and public health discussions, including analytical performance, diagnostic certainty, clinical decision-making, and long-term management strategies. To facilitate interpretation of these issues in the context of the existing evidence base, we have summarized the most relevant data and considerations in a structured Frequently Asked Questions (FAQ) section (textbox) and discuss our results further in the following sections.

### Screening feasibility and analytical performance

The German and Austrian pilot programs show that population-based NBS for MLD is feasible and is feasible in routine settings. Among 359,282 screened newborns, nine infants were identified and subsequently confirmed to have MLD, corresponding to a detection rate of 1 in 39,920. No false-positive referrals were recorded during the study period. Although the small number of affected infants limits precision estimates and long-term sensitivity will require continued surveillance, the observed detection rate aligns with the upper range of reported European birth prevalence [3]. The three-tier screening algorithm was applied across participating laboratories. Crucially, screening was performed in routine laboratories and linked to real clinical decision-making. The findings extend earlier pre-pilot and pilot data and indicate that robust analytical performance can be maintained under real-world screening conditions [13, 14].

### Diagnostic validity and avoidance of ambiguous findings

This three-tiered NBS algorithm not only minimized ambiguity in variant interpretation but also prevented incidental findings that could complicate counselling and clinical decision-making at a population level. The integration of biochemical and genetic data is a key strength of this screening approach.

All screen-positive infants showed concordant elevations in sulfatides in urine and reductions in leukocyte ARSA activity, allowing confident classification of *ARSA* variants including those previously unreported alleles within ACMG frameworks. All novel variants could be classified as likely pathogenic through biochemical confirmation.

Importantly, no individual harboring solely pseudodeficiency alleles screened positive in line with prior reports, despite their high population frequency [14]. Heterozygous carriers of variants in *ARSA*, *SUMF1*, or *PSAP* exhibited isolated sulfatide species elevations (C16:0 or C16:1-OH) in the first tier and variably reduced ARSA activity in DBS, but lacked MLD-related genotypes, and were thus appropriately filtered by the three-tier workflow as reported before [16]. In contrast, the Italian prospective screening pilot used a two-tiered approach and repeat DBS sampling to mitigate false positive referrals. This protocol resulted in additional contact with 0.02% of neonates screened, which may have been mitigated by using the three tier algorithm [17].

Conditions with overlapping biochemical signatures, such as saposin B deficiency or multiple sulfatase deficiency, were not detected as screen-positives. Due to the current lack of disease-modifying therapeutic options these biochemically related disorders need to be excluded from recall. Therefore, the inclusion of *ARSA* sequencing as a third tier was essential for excluding these disorders and for minimizing unnecessary recalls, supporting the value of a three-tier approach over purely biochemical strategies. Overall, this design limited incidental findings and avoided unnecessary interpretive challenges.

### Prediction of disease onset and biomarker limitations

Early identification of MLD through NBS necessitates reliable strategies for predicting disease onset and guiding clinical management. In this cohort, genotype-based prediction, supported by residual enzyme activity and consensus criteria, enabled stratification into early-onset and late-onset phenotypes shortly after diagnosis [19]. All infants predicted to develop late-infantile or early juvenile MLD were referred promptly for evaluation for HSPC-GT, while those predicted to develop late-onset disease entered structured surveillance programs. This stratified approach allowed timely initiation of therapy for infants at highest risk of rapid disease progression while avoiding premature intervention in those likely to remain clinically presymptomatic for years. Although genotype-based prediction cannot fully capture individual variability, its application within a standardized consensus framework proved clinically actionable and feasible in routine care settings. Additional biochemical markers used for confirmation and monitoring have important limitations. Urinary sulfatide concentrations show substantial biological and pre-analytical variability and limited utility for longitudinal assessment as they do not decrease after treatment when measured in urine. Measurement of ARSA activity in leukocytes likewise exhibit biological variability, underscoring the need for iterative measurements. However, an activity below 1% in a standardized assay is predictive for early onset [24]. Of note, we detected slightly elevated neurofilament light chain and glial fibrillary acidic protein levels in blood of predicted early-onset cases, while the levels in a child with predicted late-onset remained within normal range during follow up (unpublished data). However, data on the predictive value of these biomarkers in MLD remain limited. As demyelinating neuropathy and subtle gallbladder abnormalities could be detected in infants of all subtypes, these are considered unlikely predictors for central disease onset (Supplement, Case reports).

These findings highlight the need for complementary markers and longitudinal datasets to refine risk prediction in presymptomatic infants.

### Early clinical management and treatment feasibility

Early clinical outcomes following presymptomatic identification were encouraging but must be interpreted with caution given the short follow-up duration. Infants who underwent gene therapy during the presymptomatic period demonstrated preserved neurological examinations and age-appropriate developmental trajectories in all but one child during early follow-up, consistent with findings from controlled clinical trials [9]. Treatment delivery within the first year of life was feasible with manageable peri-transplant complications and no unexpected safety signals. However, follow-up remains limited in duration, and subtle abnormalities - including peripheral neuropathy and mild motor delay in one case - underscore that presymptomatic treatment does not necessarily prevent all disease manifestations [30]. Infants managed through surveillance for predicted late-onset disease have remained clinically asymptomatic, supporting the short-term safety of a monitoring-based approach when guided by consensus recommendations.

### Monitoring challenges and unmet needs

Monitoring presymptomatic infants identified through NBS remains a major challenge. While urinary sulfatides are indispensable for diagnosis, their longitudinal utility for monitoring is limited by biological and pre-analytical variability. Plasma biomarkers such as neurofilament light chain and glial fibrillary acidic protein show promise as indicators of early neuroaxonal injury, but age-specific reference ranges and validated thresholds for clinical decision-making are lacking [31]. These biomarkers do not reliably distinguish between central and peripheral demyelination. This distinction is clinically important for treatment timing and post-treatment monitoring, as current therapies primarily affect central nervous system disease, whereas peripheral demyelination often continues [30]. Conventional neuroimaging was largely unremarkable during early follow-up and showed in this presymptomatic stage no correlation with predicted disease subtype. Although it was particularly pronounced in late infantile cases, peripheral demyelinating neuropathy could be detected in all subtypes within the first year of life and was not halted by HSPC-GT. These findings highlight the current absence of sensitive and reliable tools to detect early subclinical progression, particularly in infants predicted to develop late-onset disease. Longitudinal studies with larger cohorts will be essential to define biomarker trajectories, refine monitoring strategies, and optimize the timing of therapeutic intervention.

### Public health implications

Beyond individual clinical outcomes, these findings have important public health implications. The biochemical-first screening approach integrates into existing NBS laboratories and provides equitable detection across diverse populations, independent of genetic ancestry. The demonstrated feasibility of downstream care pathways from confirmatory diagnostics to specialist referral and treatment addresses a key requirement for population screening programs: that a positive NBS result leads to timely and meaningful clinical action. While broader implementation will require health-economic and policy considerations specific to each system, this pilot provides evidence that cross-border NBS-enabled care for MLD, as was done in Germany and Austria, is achievable. The timing of these findings is particularly relevant in light of recent policy developments. Norway has incorporated MLD into its national newborn-screening programme; in the United States, MLD was added to the Recommended Uniform Screening Panel (RUSP) in December 2025, with implementation remaining state-dependent; and Sweden has recommended implementation, with national introduction under preparation. These developments reflect growing international consensus on the benefit of early detection through NBS. A central concern during such policy deliberations is whether screening can be translated into timely, effective care without undue harm. The present study directly addresses this by demonstrating that NBS for MLD can be operationalized within nationwide healthcare systems and linked to meaningful early clinical outcomes.

A recent perspective article highlighted the challenges of early detection in late-onset disease [19]. In MLD, it is important to emphasize that there is no “late mild” phenotype; rather, current evidence shows that even late-onset MLD, with symptom onset ranging from school age to adulthood, is associated with severe and clinically meaningful disease burden. Nevertheless, health surveillance and decisions regarding the timing of intervention in late-onset MLD should consider the particular challenges faced by identified individuals and their families and should incorporate as well as further develop psychosocial support strategies for this unique context.

### Strengths and limitations

This work reports prospective real-world outcomes following population-based NBS for MLD, encompassing the entire pathway from population screening and confirmatory diagnostics to phenotype-based treatment stratification and early clinical follow-up. Key strengths include the large, screened population, the prospective design, the integration of standardized, consensus-based care pathways, and the linkage of screening results to real-world clinical decision-making and early outcomes across two national healthcare systems.

This study also has important limitations. First, the workflow during these pilot programs did not reflect that expected once MLD NBS is fully implemented in the public health environment. For example, the time to obtain definitive positive screen results and communicate them to the QTC Tübingen was longer and more variable than desired, based on the stage of development of the algorithm. Second, the number of affected infants is small, reflecting the rarity of MLD, which limits statistical precision and precludes formal comparisons between management strategies. Third, follow-up duration remains limited, particularly for those under surveillance for late-onset disease; consequently, long-term neurological, cognitive, and quality-of-life outcomes cannot yet be assessed. Fourth, phenotype prediction relied primarily on genotype-based criteria and residual enzyme activity, which, although supported by expert consensus, do not fully capture intrafamilial and individual variability. Finally, these findings originate from well-resourced healthcare systems with established specialist networks, which may limit generalizability to settings with fewer resources or differing care structures.

## Conclusion

These German and Austrian pilot programs demonstrate that NBS for MLD can be implemented within existing healthcare infrastructures and linked to standardized diagnostic and clinical care pathways. A three-tier screening algorithm enabled early identification of affected infants and timely clinical stratification, supporting presymptomatic intervention for early-onset disease and structured surveillance for late-onset phenotypes. During early follow-up, infants managed through these pathways showed preserved neurological and developmental status. Together, these findings provide real-world evidence that NBS-enabled care for MLD is feasible and clinically actionable. Ongoing follow-up and broader implementation studies will be essential to define long-term outcomes and inform future screening policy decisions.

**Table:** 

	**Frequently Asked Questions on NBS for MLD** **Q1: How accurate is newborn screening for MLD?** **A1:** Using a validated three-tier algorithm comprising sulfatide quantification, ARSA enzyme analysis, and ARSA gene sequencing no false-positive cases were reported in a cohort of over 359,282 newborns. Every infant identified (*n* = 9) as screen-positive was subsequently confirmed to have MLD. The approach reliably distinguishes affected individuals from carriers and pseudodeficient individuals [14] as well as biochemically mimicking disorders, underscoring its screening precision. Although the sensitivity cannot be assessed in a prospective screening setting, data from multiple retrospective and prospective pilots (Germany, Italy) have all reported 100% detection of known MLD DBS samples [13–17]. Robust quality control protocols and standardized methods are published to ensure consistency across screening labs [15, 21] **Q2: How are uncertain genetic results handled in MLD screening?** **A2:** The structured three-tier screening strategy minimizes diagnostic ambiguity. *ARSA* variants of uncertain significance (VUS) are rare due to the available functional validation using biochemical data (sulfatide levels and ARSA activity in leukocytes). Based on current databases and registries most of clinically relevant *ARSA* variants detected in MLD patients with European ethnic background are reported in literature. In the rare instance of a novel *ARSA* variant, elevated sulfatides in urine and low enzyme activity in leukocytes provide strong functional evidence to reclassify VUS into likely pathogenic/pathogenic or likely benign/benign categories. Besides, several easily accessible in vitro and complementary in silico models are established to complement routine assay and prove pathogenicity of novel variants [24, 32]. To date, no screen-positive infant has remained genetically uncertain, and all screen-positive cases were confirmed as true MLD diagnoses **Q3: Could screen-positive results reflect non-MLD disorders or benign MLD forms?** **A3:** No. All screen-positive infants in the pilot exhibited persistent biochemical abnormalities and ARSA genotypes consistent with MLD. Due to the specificity of the biochemical first- and second-tier assays, functionally benign ARSA genotypes i.e., those not associated with disease causing enzyme deficiency, are not expected to be identified as screen-positive. Within the spectrum of MLD, although the age of onset may vary, there are no non-MLD- phenotypes; all affected individuals inevitably experience progressive neurological deterioration leading to premature death. ARSA pseudodeficiency does not lead to elevated sulfatides and will thus not be flagged in the first tier as shown by Wu and colleagues [14]. Carriers will be excluded in the third tier based on genetic testing. Potential biochemical mimics, such as saposin B or multiple sulfatase deficiencies, were not observed in this case series, however, carriers were identified [16] Importantly, the inclusion of *ARSA, PSAP* and *SUMF1* gene sequencing in the panel enables differentiation from these mimicking disorders, so that these patients are currently excluded from recalls due to the absence of effective therapeutic options **Q4: Why is genetic testing a necessary third tier in MLD screening?** **A4:** Genetic testing is essential to distinguish true positives from mimicking disorders and carriers, who may exhibit biochemical abnormalities in the first and second tiers. The inclusion of *ARSA* sequencing detects clinically relevant *ARSA* variants, ensuring accurate diagnosis and avoiding unnecessary recalls. Another pilot studies using only biochemical testing has demonstrated higher recall rates and reduced specificity [17], reinforcing the necessity of an integrated third tier. Furthermore, mimicking conditions such as saposin B deficiency and multiple sulfatase deficiency can and ethically should be excluded from recall procedures in the context of NBS, because no effective treatments currently exist for these disorders [20] **Q5: Can we predict the disease onset for all presymptomatic newborns with MLD?** **A5:** In all of the nine newborns identified in our pilot programs, the underlying ARSA genotype provided reliable guidance on the likely age of disease onset. This is not unexpected, given that most *ARSA* gene variants are well-characterized and strongly associated with specific clinical subtypes. In addition, residual ARSA enzyme activity in leukocytes serves as biochemical indicator for very early-onset forms of the disease [24] The challenge, however, remains in particular to predict late-onset and uncertain subtypes. Therefore, for the small subset of newborns with uncertain or novel genotypes, the European consensus on clinical monitoring is in place to guide surveillance and ensure timely intervention if needed [20]. As research progresses, including efforts to refine genotype-phenotype models and expand biomarker use, the ability to predict disease onset with even greater accuracy is expected to improve **Q6: How are infants with predicted late-onset MLD managed?** **A6:** Infants identified with genotypes linked to late-onset forms are enrolled in structured surveillance programs according to the European consensus recommendations [20]. These include periodic clinical assessments, developmental evaluations, and biomarker monitoring. This conservative management approach is informed by consensus recommendations and balances early identification with proportional intervention. Families are supported by multidisciplinary teams, and HSCT is planned individually during the pre-school period, with timing determined by genotype, age and emerging clinical, imaging, neurophysiological or biomarker evidence; predefined changes may prompt earlier treatment reassessment. To date, families have responded positively to this anticipatory care model. We also acknowledge the uncertainty surrounding the optimal timing of intervention for late-onset MLD. As discussed above, care for these individuals and their families must be further improved through more structured surveillance, better-supported clinical decision-making, and enhanced psychosocial support **Q7: Is it ethically justified to screen all newborns for MLD?** **A7:** Yes. Given that we now have effective presymptomatic treatment options for early (HSPC-GT) and late onset (HSCT) that can alter the otherwise progressive disease course of MLD, it is ethically compelling to identify affected infants as early as possible. Recent long-term follow-up data continue to underscore that the earlier the intervention the better the outcome [9]. Also for late onset MLD, data reveal an improved outcome if HSCT is performed in the presymptomatic disease stage [7] Hence NBS for MLD satisfies the established ethical and public health principles for NBS, as outlined by Wilson and Jungner [33]. MLD is a severe, progressive, and ultimately fatal neurodegenerative disorder if left untreated Failure to implement screening would result in continued diagnoses only after the onset of irreversible neurological damage, except cases with a positive family history, thereby depriving index cases of the opportunity to pursue effective early treatment [11]. Early detection also mitigates the prolonged uncertainty and psychological distress associated with delayed diagnosis, enabling timely, informed clinical and reproductive decision-making. Furthermore, screening promotes equity in healthcare by ensuring that all newborns, not only those with a known family history or incidental early diagnosis, have the opportunity to benefit from life-saving interventions [11, 34] **Q8: Is newborn screening for MLD cost-effective and sustainable?** **A8:** Emerging health economic analyses indicate that adding MLD to NBS is cost-effective. A UK study showed that NBS for MLD provides a favorable cost per quality-adjusted life year (QALY) gained, well within typical thresholds for healthcare interventions [35–37]. While HSPC-GT has a high upfront cost, it has been found cost-effective in both the UK and US settings, especially when given presymptomatically. This is because treating early prevents the enormous lifetime costs of unmanaged MLD which include intensive medical care, disability support, and lost productivity. In the long term, preventing even a few cases of severe MLD through NBS can save healthcare systems millions of Euros in downstream expenses [38]. As more regions implement MLD screening worldwide, economies of scale and shared best practices will further improve cost-efficiency. **Q9: Are we ready to implement MLD screening nationally in terms of infrastructure and expertise?** A9: The pilots demonstrate substantial readiness, although national implementation still requires jurisdiction-specific validation, governance, funding and treatment capacity. The pilot programs have effectively paved the way – the laboratory protocol for MLD screening has been optimized and published as consensus SOPs and Norway has incorporated MLD into its national programme, the US added MLD to the RUSP in December 2025 (with state-level implementation ongoing), and Sweden has recommended national introduction. This means national programmes can build on a well-characterised template: the MS/MS assay reagents and methods are available, and training can be obtained from experienced centers. On the clinical side, treatment centers for MLD (HSPC-GT/HSCT) are operational, and recommendations for confirmatory testing, follow-up, and treatment have been developed by expert consortia [20]. The German-Austrian pilot demonstrated that coordination between the screening lab and a specialized treatment center can be well coordinated. We have built referral networks, case management protocols, and educational materials for clinicians and families – all of which can be scaled up to a national level. Importantly, international collaborations (MLDi) are in place to support new programs with technical and clinical expertise. Remaining steps include jurisdiction-specific validation, governance, securing approval and funding, and ensuring treatment capacity before formally including MLD in the national screening panel. Once that is in place, implementation can proceed quickly with the pilot experience serving as a detailed roadmap

## Electronic supplementary material

Below is the link to the electronic supplementary material.

Supplementary material 1

## Data Availability

No datasets were generated or analysed during the current study.

## Abbreviations

ACMG: American College of Medical Genetics and Genomics
ARSA: Arylsulfatase A
CNS: Central nervous system
DBS: Dried blood spot
EJ: Early juvenile
EMA: European Medicines Agency
HSPC-GT: Haematopoietic stem and progenitor cell gene therapy
HSCT: Haematopoietic stem cell transplantation
LI: Late infantile
LJ: Late juvenile
LS-MS/MS: Liquid chromatography–tandem mass spectrometry
MLD: Metachromatic leukodystrophy
MLDi: Metachromatic Leukodystrophy Initiative
MRI: Magnetic resonance imaging
MSD: Multiple sulfatase deficiency
NBS: Newborn screening
NCV: Nerve conduction velocity
NGS: Next-generation sequencing
pPTV: Predicted protein-truncating variant
*PSAP*: Prosaposin gene
QTC: Qualified treatment center
RUSP: Recommended Uniform Screening Panel
*SUMF1*: Sulfatase-modifying factor 1 gene
VUS: Variant of uncertain significance
