# Supplementary material for: Clinical management of metachromatic leukodystrophy cases identified in the German and Austrian Newborn screening pilots
Source: Orphanet J Rare Dis. 2026 Oct 4;21:345. doi: 10.1186/s13023-026-04620-y (PMC13640125; doi:10.1186/s13023-026-04620-y)
Supplement: Supplementary file 1 [file 13023_2026_4620_MOESM1_ESM.docx]

Supplementary Materials

1. Methods

1.1 Detailed Screening Algorithm and Workflow

A three-tier biochemical and molecular screening algorithm was employed, based on international consensus protocols.^1^ NBS for MLD was conducted within the Archimed*life* network and the Hannover Screening Laboratory, Germany, between November 2021 and July 2025. Screening protocols were implemented in consecutive phases to optimize analytical workflows.

In tier 1, dried blood spots (DBS) collected within 48 to 72 hours postnatally were analysed for C16:0 and C16:1-OH sulfatides using liquid chromatography with tandem mass spectrometry (LC-MS/MS).^1-3^ Analytes were extracted from a single 3.2 mm punch and quantified using an isotopically labelled internal standard (d₅-C16:0 sulfatide). Results were reported as absolute concentrations (µmol/L) and multiples of the median (MoM) for the birth population. Second-tier testing quantified ARSA enzymatic activity in DBS eluates using LC-MS/MS with a synthetic sulfatide analog substrate and internal standard.^1,3,4^ During the initial pilot phase, ARSA activity was assessed in parallel with sequencing due to technical challenges and to validate the assay.^5^

Third tier testing involved molecular analysis of the *ARSA* gene to confirm the presence of presumably bi-allelic, clinically relevant variants. DNA was eluted from the DBS and next-generation sequencing (NGS) was performed targeting the *ARSA* gene. The protocols also included sequencing of the *SUMF1* gene (to rule out multiple sulfatase deficiency) and *PSAP* gene to rule out Saposin B deficiency), given that these rare conditions can phenocopy MLD biochemically. Pathogenicity of *ARSA* variants was evaluated according to ACMG guidelines.^6^ Notably, if single *ARSA* variants were found, the corresponding infant would be considered a carrier, not a screen positive and hence not receive a recall. Conversely, infants with one homozygous or two potentially compound-heterozygous clinically relevant *ARSA* variants were considered screen positive for MLD.

**2. Results**

### **Table S1. Clinical Assessment**

|  | **Index 1** | **Index 2** | **Index 3** | **Index 4** | **Index 5** | **Index 6** | **Index 7** | **Index 8** | **Index 9** |
| --- | --- | --- | --- | --- | --- | --- | --- | --- | --- |
| **Predicted Subtype** | **Early juvenile** | **Early juvenile** | **Late onset** | **Late infantile** | **Late infantile** | **Late infantile** | **Early juvenile** | **Late infantile** | **Late onset** |
| **Pregnancy and delivery** | Full term, unremarkable | Full term, unremarkable | 34+0 gestational weeks | Full term, unremarkable | Full Term, unremarkable | Full Term, unremarkable | Full term, unremarkable | Full term, unremarkable | Full term, unremarkable |
| **NBS result**  **[age]** | 2 months | 3 months | 3 months | 3 months | 3 months | 6 months | 3 months | 5 months | 1 month |
| **QTC_Referral_ [age]** | [3 months] | [4 months] | [4 months] | [4 months] | [7 months] | [8 months] | [4 months] | [7 months} | [5 months] |
| **Diagnosis of MLD confirmed**  **[age]** | [2 months] | [3 months] | [3 months] | [3 months] | [5 months] | [6 months] | [4 months] | [5 months] | [1 month] |
| **Neuropediatric examination at QTC**  **[age]** | Craniosynostosis, otherwise unremarkable  [3 and 9 months] | Unremarkable  [4 and 10 months] | Unremarkable  [4 and 8 months] | Unremarkable  [3 months; 5 months] | Unremarkable  [6 months] | slight motor delay [5 months]  Unremarkable  [7 months] | Unremarkable  [4 months] | Unremarkable [5 months] | Unremarkable,  [5 months] |
| **Brain MRI [age]**  **[MRI-MLD score]** | Unremarkable [9 months]  [MRI-MLD score 0] | Unremarkable [10 months]  [MRI-MLD score 0] | Unremarkable [8 months]  [MRI-MLD score 0]  Unremarkable [20 months]  [MRI-MLD score 0]  Unremarkable [31 months]  [MRI-MLD score 0] | Unremarkable [5 months]  [MRI-MLD score 0] | Unremarkable [8 months]  [MRI-MLD score 0] | Unremarkable  [6 months] [MRI-MLD score 0] | Unremarkable  [7 months]  [MRI-MLD score 0] | Unremarkable  [7 months]  [MRI-MLD score 0] | Scheduled |
| **Nerve conduction studies**  **[age]** | Demyelinating neuropathy  [9 months] | Demyelinating neuropathy  [10 months] | Demyelinating neuropathy  [8 months, 20 months, 31 months] | No pathological appearance [5 months] | Mild demyelinating neuropathy [6 months] | No pathological appearance [6 months] | Missing F-wave,  [7 months]  Demyelinating neuropathy  [12 months] | Early demyelinating neuropathy  [7 months] | Scheduled |
| **Gallbladder ultrasound**  **[age]** | Unremarkable  [9 months] | Unremarkable  [10 months] | Unremarkable  [8 months], 20 months and 31 months] | Slightly tick walled  [5 and 7 months] | Unremarkable  [6 months] | Unremarkable  [11 months] | Unremarkable  [9 months] | Evidence of Sludge  [7 months] | Unremarkable  [5 months] |
| **Age at treatment decision** | 3 months | 5 months | 4 months | 4 months | 5 months | 6 months | 7 months | 6 months | 3 months |
| **Apheresis** | Apheresis  [10 months] | Apheresis  [11 months] | n.a. | Apheresis  [6 months] | Apheresis  [7 months] | Apheresis  [9 months] | Apheresis in planning | Apheresis1  [7 months]  Apheresis 2  [9 months] | n.a. |
| **Treatment**  **[age]** | arsa-cel infusion  [12 months] | arsa-cel [13 months] | HSCT scheduled [2 to 5 years] | arsa-cel infusion  [8 months] | arsa-cel infusion  [9 months] | arsa-cel infusion  [11 months] | arsa-cel infusion in planning | arsa-cel infusion scheduled | HSCT scheduled [2 to 5 years] |
| **MRI follow-up**  **[age]**  **[MRI-MLD score]** | Unremarkable [19 months] [MRI-MLD score 0]  Unremarkable [24 months]  [MRI-MLD score 1]  Unremarkable  [36 months]  [MRI-MLD score 2] | Unremarkable [19 months] [MRI-MLD score 0]  Unremarkable [25 months]  [MRI-MLD score 0] |  | Unremarkable [14 months][MRI-MLD score 0]  Unremarkable [19 months]  [MRI-MLD score 0] |  |  |  |  |  |
| **Nerve conduction studies**  **[age]** | Improved, demyelinating neuropathy [19 months]  Improved demyelinating [24 months]  Unchanged [36 months] | Deteriorating demyelinating neuropathy [25 months] |  | Deteriorating demyelinating neuropathy [14 months]  Deterioration, demyelinating neuropathy  [20 months,] |  |  |  |  |  |
| **Gallbladder ultrasound**  **[age]** | Unremarkable [18 months]  Unremarkable  [24 months] | Wall slightly thickened (1.3 mm) [19 months]  Unremarkable [25 months] |  | Deterioration, thickened gallbladder wall (3 mm) [20 months] | Slight sludge  [9 months]  Unremarkable  [10 months] | Unremarkable  [11 months] |  |  |  |
| **Last Follow-up at QTC [age]** | 37 months | 34 months | 32 months | 20 months | 15 months | 17 months | 9 months | 8 months | 5 months |
| **Neuropediatric evaluation at last Follow-up** | Age appropriate | Age appropriate | Age appropriate | Mild developmental delay in motor skills | Age appropriate | Age appropriate | Age appropriate | Age appropriate | Age appropriate |
| **Current age** | 42 months | 38 months | 37 months | 26 months | 16 months | 18 months | 15 months | 12 months | 8 months |

**Supplementary Table S2:**

| Event | mean | SD | median | count | range |
| --- | --- | --- | --- | --- | --- |
| Age at NBS Result [months] | 3.2 | 1.4 | 3.0 | 9 | 1.0 - 5.8 |
| Age at Confirmation of Diagnosis [months] | 3.7 | 1.5 | 3.4 | 9 | 1.1 - 6.3 |
| Age at Referral to QTC [months] | 5.2 | 1.9 | 4.1 | 8 | 3.4 - 8.3 |
| Age at Decision for Treatment [months] | 5.1 | 1.3 | 5.1 | 8 | 3.3 – 6.9 |
| Age at Apheresis [months] | 8.3 | 1.9 | 8.0 | 6 | 5.9 - 10.9 |
| Age at Second Apheresis [months] | 8.9 |  | 8.9 | 1 | 8.9 – 8.9 |
| Age at Conditioning [months] | 10.5 | 2.2 | 10.8 | 5 | 7.6 - 13.2 |
| Age at Infusion [months] | 10.7 | 2.2 | 11.0 | 5 | 7.8 - 13.4 |
| Age at Discharge [months] | 11.7 | 2.3 | 11.8 | 5 | 8.6 - 14.7 |
| Age at 1st Neurological Follow-up [months] | 17.1 | 2.0 | 17.2 | 5 | 14.9 - 19.3 |
| Age at 2nd Neurological Follow-up [months] | 23.1 | 2.9 | 24.2 | 3 | 19.7 - 25.2 |
| Age at last Follow-Up in Tübingen [months] | 21.4 | 11.3 | 18.5 | 8 | 7.5 - 36.7 |
| Current Age [months] | 23.7 | 12.7 | 18.5 | 9 | 7.9 – 42.0 |

2.2 *Case reports*

**Index 1 – Early-Juvenile MLD (current age: 3 years 6 months):**

Index 1 was a term-born male infant with normal perinatal history and unremarkable early development. NBS results were available at the age of 2.2 months. Residual ARSA enzyme activity was markedly reduced, consistent with an early-juvenile phenotype associated with the genotype *ARSA*: c.[412C>T];[1283C>T],p.[(Pro138Ser)];[(Pro428Leu)]. At last evaluation before HSPC-GT at 9 months of age, nerve conduction studies (NCS) demonstrated a demyelinating neuropathy characterized by prolonged minimal F-wave latencies and reduced motor conduction velocity, while brain MRI and nerve ultrasound were unremarkable.

The patient underwent HSPC-GT at 11.8 months of age. Post-treatment, ARSA activity in leukocytes rose to supraphysiological levels (Figure S1). Follow-up MRI examinations remained normal. NCS showed stable, but significant demyelinating neuropathy, and nerve ultrasound revealed progressive nerve enlargement. At the latest follow-up, the child demonstrated normal neurodevelopment, independent ambulation, and no radiological or electrophysiological signs of disease progression. Sitting without support was documented at 9.7 months, standing without support at 11 months, and independent walking at 15 months. First words were documented at 9.7 months, and two-word sentences at 12 months, according to medical records. At the age of 3 years, cognitive assessment using the WPPSI-IV revealed a full-scale IQ of 108. Motor function was assessed using the GMFC-MLD score at 19, 24, and 36 months of age and was 0 at all time points.

**Index 2 – Early-Juvenile MLD (current age: 3 years 2 month):**

Index 2 was a term-born male infant with normal perinatal history and development. NBS results were available 3.1 months after birth. The genotype *ARSA*: c.[465+1G>A];[1283C>T],p.[(?)];[(Pro428Leu)] and low residual enzyme activity supported an early-juvenile phenotype. At 11 months, NCS showed a demyelinating neuropathy, and AEPs revealed bilateral prolongation of interpeak latencies III–V, consistent with central auditory pathway involvement. Abdominal ultrasound demonstrated an unremarkable gallbladder wall.

The patient underwent arsa-cel therapy at 13.4 months of age, leading to an increase in ARSA activity to supraphysiological levels. Adverse events not related to HSPC-GT included Hickman catheter–associated *Staphylococcus aureus* sepsis, which required catheter removal and reimplantation prior to transplantation, as well as a thrombus of the right external iliac vein related to apheresis.

Serial MRI scans remained unremarkable. Gallbladder wall thickening resolved, and NCS showed stable findings, while AEPs continued to show mild latency prolongation without functional impact.

At the most recent follow-up, neurodevelopment was age appropriate. The child remains symptom free with normal motor development and no evidence of disease progression. Standing without support was documented at 12 months and walking without support at 19 months. First words were documented at 12 months. Cognitive assessment at 25 months of age using the Bayley Scales of Infant and Toddler Development, Third Edition (Bayley-III), revealed a cognitive scaled score of 90. At 37 months of age, assessment with the SON-R 2–8 showed a full-scale IQ of 105. Motor function was assessed using the GMFC-MLD score at 19, 25, 34 and 37 months of age and was 0 at all time points.

**Index 3 – Late-Onset MLD (current age: 3 years 1 months):**

Index 3 was a male infant born at 34 weeks’ gestation with a prenatally diagnosed omphalocele, surgically corrected postnatally. After a positive MLD newborn-screening result at 3.0 months after birth, genetic testing confirmed the genotype *ARSA*: c.[465+1G>A];[542T>G],p.[(?)];[(Ile181Ser)], a genotype previously associated with late-juvenile and adult-onset MLD.

Evaluation at 8 months revealed demyelinating changes on NCS with prolonged F-wave latency and reduced motor conduction velocities, as well as AEPs showing delayed wave I on the right and delayed waves III and V bilaterally, indicating combined peripheral and central auditory involvement. MRI at that time was unremarkable.

Follow-up MRI demonstrated age-appropriate myelination, and NCS findings remained consistent with mild, non-progressive peripheral neuropathy. Nerve ultrasound showed no enlargement.

At the latest follow-up, neurodevelopment was normal with age-appropriate cognitive and motor milestones. The patient remains clinically asymptomatic under annual neurological surveillance, with hematopoietic stem cell transplantation planned between 2 and 5 years of age. The patient started walking at 18 months and began speaking single words. Multiword sentences were documented at 31 months. Cognitive assessment with the Bayley-III at 32 months revealed a cognitive scaled score of 115. GMFC-MLD remained 0 and was last assed at 32 months of age.

**Index 4 – Late-Infantile MLD (current age: 2 year 2 months):**

Index 4 was a term-born male infant with normal early development. NBS results were available at the age of 2.7 months. The patient was homozygous for the canonical splice-site variant *ARSA*: c.[465+1G>A];[465+1G>A],p.[(?)];[(?)], a well-established loss-of-function genotype associated with late-infantile MLD. Residual ARSA enzyme activity was below the early-onset threshold. At 6 months, gallbladder ultrasound revealed mild wall thickening (3 mm) without sludge, whereas MRI and NCS were unremarkable. Following arsa-cel therapy at 8 months, the post-conditioning course was complicated by hepatic veno-occlusive disease, which resolved with defibrotide treatment and supportive care. ARSA activity increased to normal-to-supraphysiological levels. Follow-up MRI demonstrated progressive, age-appropriate myelination without pathology. NCS revealed a clearly progressive demyelinating polyneuropathy compared with baseline, while nerve ultrasound showed no enlargement. At the latest follow-up, the patient remained clinically stable with normal cognitive development, but mild developmental motor delay despite no radiological evidence of central disease progression. The patient began attempting to crawl and sit independently at 15 months and walked with support at 19.7 months. The first word was documented at 15 months. Motor function assessed via GMFC-MLD was 2 at 19.7 months. Cognitive assessment with the Bayley-III at 19.7 months revealed a scaled score of 80 in the cognitive domain.

**Index 5 – Late-Infantile MLD (current age: 1 year 4 months):**

Index 5 was a male infant, the second child in the family, with normal perinatal course and development. NBS results were available at 2.9 months after birth. Genetic testing showed compound heterozygosity for two protein-truncating variants, *ARSA*:c.[465+1G>A];[899del], p.[(?)];[(Leu300Cysfs*29)]. The splice-site variant is a known loss-of-function allele, and the frameshift variant is novel but predicted to result in a truncated, nonfunctional protein. Residual leukocyte ARSA activity was nearly undetectable, consistent with late-infantile MLD.

At 6 months, NCS demonstrated demyelinating features with prolonged minimal F-wave latency and reduced motor conduction velocity in the right tibial nerve, whereas MRI was unremarkable.

Following stem-cell collection and arsa-cel infusion at age 9.4 months, leukocyte ARSA activity increased to normal-to-supraphysiological levels. The patient remains clinically and neurologically stable with normal development. The patient was able to stand independently and walk with support at 15 months. First words were documented at 15 months. Motor function assessed at 7 months was age appropriate. Cognitive assessment with the Bayley-III at 15 months revealed a scaled score of 95 in the cognitive domain.

**Index 6 – Late-Infantile MLD (current age: 1 year 6 months):**

Index 6 was a full-term female infant, the second child of consanguineous parents. NBS results were available at 5.8 months after birth.

MRI and NCS were initially normal, and gallbladder ultrasound was unremarkable.

The genotype was *ARSA:*c.[931G>A];[931G>A], p.[(Gly311Ser)];[(Gly311Ser)], a missense variant associated with late-infantile MLD. Residual ARSA activity was markedly reduced. The patient underwent arsa-cel infusion at 11 months of age, resulting in normal-to-supraphysiological enzyme activity. Adverse events included Hickman catheter–associated *Staphylococcus epidermidis* infection, as well as Grade I allergic reactions to medications during central venous catheter placement for apheresis.

Neurodevelopment and MRI findings remained normal under follow-up. The patient achieved independent sitting at 7.4 months and independent walking at 15 months. Single words were spoken at 17 months. Cognitive assessment with the Bayley-III at 17 months revealed a scaled score of 75 in the cognitive domain.

**Index 7 – Early-Juvenile MLD (current age: 1 year 3 month):**

Index 7 was a term-born male infant with normal perinatal history and early development. NBS results were available at the age of 3.3 months. Genetic testing identified *ARSA*:c.[465+1G>A];[494C>T], p.[(?)];[(Pro165Leu)]. The splice-site variant is pathogenic, whereas the missense variant is of uncertain significance but, *in trans*, predicts early-juvenile onset; on the basis of the concordant biochemical profile, the variant was subsequently reclassified as likely pathogenic. Residual ARSA activity was moderately reduced. An early-juvenile phenotype was predicted based on the genotype including a common pPTV.

At 7 months, NCS revealed absent F-waves in the median nerve but was otherwise normal; MRI and nerve ultrasound were unremarkable, and gallbladder imaging was normal. During a second evaluation at the age of 12 months NCS revealed clearly progressing demyelinating neuropathy. The patient is scheduled for apheresis followed by HSPC-GT at the time of writing, following repeat multidisciplinary panel review. Development remained age-appropriate.

**Index 8 – Late-Infantile MLD (current age: 12 months):**

Index 8 was a female infant born at 39 weeks’ gestation with normal perinatal history. NBS results were available 4.7 months after birth. The genotype was *ARSA*:c.[684+1G>A];[1223_1231del], p.[(?)];[(Val408Serfs*21)], comprising two truncating variants predicted to abolish enzyme function. Residual ARSA activity was undetectable, consistent with late-infantile MLD.

Neurological evaluation at 7 months was age-appropriate. MRI showed slight frontal and parietal white-matter hyperintensities attributed to incomplete myelination. NCS revealed early demyelinating polyneuropathy with prolonged distal motor latency of the median nerve and reduced conduction velocity in the tibial nerve. Gallbladder ultrasound demonstrated sludge, while nerve ultrasound was normal. Two apheresis procedures were performed, and arsa-cel infusion is planned. The patient remains clinically stable under close follow-up. Sitting without support was documented at the age of 8.7 months. Motor function assessed at 7 months was age appropriate. Cognitive assessment with the Bayley-III at 7 months revealed a scaled score of 110 in the cognitive domain. Adverse events including Hickman catheter dislocation requiring repositioning and reimplantation, and hypokalaemia during both apheresis procedures.

**Index 9 – Late-Onset MLD (current age: 8 months):**

Index 9 was a term-born female infant with normal pregnancy, delivery, and early development. NBS results were available 1.0 months after birth. Genetic testing revealed homozygosity for *ARSA:*c.[1283C>T];[1283C>T], p.[(Pro428Leu)];[(Pro428Leu)], a prevalent European allele retaining partial enzyme activity. Leukocyte ARSA activity was reduced but above the early-onset threshold. A late-onset phenotype was predicted, with expected symptom onset after 6 years of age.^7,8^

The patient is currently under annual neurological surveillance, with HSCT planned between 2 and 5 years. Neurodevelopment remains normal, and no abnormalities have been detected.

**Figure S1.** Longitudinal ARSA enzyme activity in leukocytes before and after treatment, with the shaded area indicating the control mean ± 3 standard deviations; control mean 3.8, SD 0.765, normal range: 1.5 to 6.1 E_514_ nm/10^6^ cells; the y-axis is split to display low and high activity ranges. Data points represent individual patients with the vertical dashed line marking the time of treatment administration. LI, late infantile; EJ, early juvenile, LO, late onset.

1.	Shaff A, Basheeruddin K, Bekri S, et al. Newborn screening for metachromatic leukodystrophy: Preparation of reagents and methodology for measurement of sulfatides and arylsulfatase A enzymatic activity in dried blood spots. *Mol Genet Metab* 2025; **145**(3): 109138.

2.	Bekri S, Bley A, Brown HA, et al. Higher precision, first tier newborn screening for metachromatic leukodystrophy using 16:1-OH-sulfatide. *Mol Genet Metab* 2024; **142**(1): 108436.

3.	Hong X, Daiker J, Sadilek M, et al. Toward newborn screening of metachromatic leukodystrophy: results from analysis of over 27,000 newborn dried blood spots. *Genet Med* 2021; **23**(3): 555-61.

4.	Hong X, Kumar AB, Daiker J, et al. Leukocyte and Dried Blood Spot Arylsulfatase A Assay by Tandem Mass Spectrometry. *Anal Chem* 2020; **92**(9): 6341-8.

5.	Laugwitz L, Mechtler TP, Janzen N, et al. Newborn Screening and Presymptomatic Treatment of Metachromatic Leukodystrophy. *N Engl J Med* 2024; **391**(13): 1256-8.

6.	Richards S, Aziz N, Bale S, et al. Standards and guidelines for the interpretation of sequence variants: a joint consensus recommendation of the American College of Medical Genetics and Genomics and the Association for Molecular Pathology. *Genet Med* 2015; **17**(5): 405-24.

7.	Santhanakumaran V, Groeschel S, Harzer K, et al. Predicting clinical phenotypes of metachromatic leukodystrophy based on the arylsulfatase A activity and the ARSA genotype? - Chances and challenges. *Mol Genet Metab* 2022; **137**(3): 273-82.

8.	Laugwitz L, Schoenmakers DH, Adang LA, et al. Newborn screening in metachromatic leukodystrophy – European consensus-based recommendations on clinical management. *Eur J Paediatr Neurol* 2024; **49**: 141–54.
